# Gender Differences in Rheumatoid Arthritis: Interleukin-4 Plays an Important Role

**DOI:** 10.1155/2020/4121524

**Published:** 2020-12-28

**Authors:** Chaojie Yu, Chong Liu, Jie Jiang, Hao Li, Jiarui Chen, Tianyou Chen, Xinli Zhan

**Affiliations:** ^1^Guangxi Medical University, Nanning 530021, China; ^2^Spine and Osteopathy Ward, The First Affiliated Hospital of Guangxi Medical University, Nanning 530021, China

## Abstract

**Introduction:**

Rheumatoid arthritis (RA) is a chronic inflammatory disease characterized by symmetrical peripheral polyarthritis. A large number of studies have shown that RA is characterized by gender differences in clinical manifestations. The purpose of this study is to identify the key molecules of gender differences in patients with RA and to provide new molecular targets for personalized therapy. *Material and Methods*. The data from GSE55457 were downloaded from the comprehensive gene expression comprehensive database, and two groups (RA vs. No-RA groups, Male-RA vs. Female-RA groups) of differentially expressed genes (EDGs) were obtained by GEO2R. The GO function and KEGG pathway analyses of DEGs were carried out through the plug-in ClueGO in Cytoscape. Based on the STRING online, a protein-protein interaction (PPI) network was constructed. Hub genes were selected from CytoHubba. Through the intersection of the top 10 hub genes in two sets of EDGs, the key genes and related KEGG pathways were found. Quantitative Real-Time PCR and Western blotting analysis were performed for verification.

**Results:**

1230 DEGs were screened between RA and No-RA groups, and 306 DEGs were screened between male and female RA groups. The common key gene of the two groups is IL-4. Between RA group and No-RA group, interleukin-4 (IL-4) participates in cytokine-cytokine receptor interaction, Th1 and Th2 cell differentiation, Th17 cell differentiation, T cell receptor signaling pathway, etc.

**Conclusion:**

This study contributes to the molecular biological mechanism of gender differences in RA. IL-4 may have played an important role.

## 1. Introduction

Rheumatoid arthritis (RA) is a chronic inflammatory disease characterized by symmetrical peripheral polyarthritis, which can be accompanied by extensive joint destruction, pulmonary lesions, cardiovascular disease, meningitis, etc. [1–5]. Chronic synovitis is one of the main pathological features of RA joint damage [2, 6]. Long-term intra-articular inflammation will lead to the destruction of articular cartilage, making patients feel chronic pain. In the late stages of the disease, disability severely reduces the quality of life of patients [7–9]. Previous studies on the pathogenesis of rheumatoid arthritis involved smoking, heredity, intestinal bacteria, and environmental factors [10–12]. Heredity is one of the susceptible factors of rheumatoid arthritis and is considered to be an endogenous promoter [11, 12]. The clinical symptoms of rheumatoid arthritis may be associated with abnormal polygene expression, but key target genes have not been found. The pathogenesis of rheumatoid arthritis has not been fully elucidated, and there is no radical cure at present.

Some research have shown that the disease was more common in women [13, 14]. According to Kvien et al. [15], the incidence of RA in women was 4-5 times higher than that in men in people under 50 years of age, and the ratio of women to men in people over 60-70 years old was about 1 : 2. Men and women had different therapeutic effects. Albrecht [16] found that there were gender differences in the prevalence of RA complications. Women are more likely to develop depression, fibromyalgia, and hypothyroidism than men, while men are more common in cardiovascular disease and diabetes. More studies have shown that women are more affected by the disease [17–20]. There were significant differences in clinical manifestations and therapeutic effects among different genders of RA [15–22]. However, it is not clear why RA has these gender differences.

There have been no previous studies on the gender differentially expressed genes (DEGs) in patients with RA. The study of key genes is helpful to explore the potential mechanism of the different characteristics of RA between sexes. We use a variety of bioinformatics methods to analyze the data in GSE55457. The purpose of this study is to reveal the key molecules of gender differences in RA patients, which is conducive to a further comprehensive understanding of the biological characteristics of RA and provide a new molecular target for personalized therapy.

## 2. Methods and Materials

### 2.1. Data Source

The data of GSE55457 were downloaded from the Gene Expression Omnibus (GEO) database (http://www.ncbi.nlm.nih.gov/geo/). GSE55457 (platform: GPL96; Affymetrix Human Genome U133A Array) detected the gene expression profile in synovium samples of the knee joint from 13 RA samples (3 males and 10 females) and 10 normal samples (8 males and 2 females). All specimens were divided into the RA group and No-RA group. Patients with rheumatoid arthritis were further divided into the male rheumatoid arthritis (M-RA) group and female rheumatoid arthritis (F-RA) group. The data are analyzed in the following order. Our research is based on data from open databases. Neither morality nor patient consent applies.

### 2.2. Data Processing

GEO2R (https://www.ncbi.nlm.nih.gov/geo/geo2r/), as an online analysis tool, can be used to filter DEGs between the RA and No-RA groups and DEGs between the M-RA and F-RA groups, respectively, [23]. The setting conditions of DEGs are *P* < 0.05 and ∣log fold‐change (FC) | >1.

### 2.3. Enrichment Analysis of GO and KEGG

The 2 DEGs were, respectively, analyzed by the plugin ClueGO [24] in Cytoscape software (version 3.6.1) [25] for Gene Ontology (GO) and Kyoto Encyclopedia of Genes and Genomes (KEGG) enrichment. The top 5 results of the analysis are described on the ImageGP website (http://www.ehbio.com/ImageGP/index.php/Home/Index/Volcanoplot.html).

### 2.4. Construction of PPI Network

We integrate the DEGs into the protein-protein interaction (PPI) network separately and use STRING [26](version 11.0) to evaluate the interaction between DEGs. A composite score > 0.4 is considered to be a statistically significant interaction. PPI network data were loaded into Cytoscape (version: 3.6.1) software for visual adjustment.

### 2.5. Identification of Key Gene

Through the plug-in Cytohubba [27] (version 0.1) in the Cytoscape software, the top 10 hub genes of the 2 PPI networks can be identified from the PPI network. The intersection of the top 10 hub genes is the key gene. Its associated pathways are found and analyzed in the KEGG pathways.

### 2.6. Quantitative Real-Time PCR (QPCR)

Synovium samples were collected and divided into the M-RA group (*n* = 3), F-RA group (*n* = 10), male No-RA group (*n* = 10), and female No-RA group (*n* = 10). All experiments were approved by the Ethics Review Committee of the First Affiliated Hospital of Guangxi Medical University.

According to the manufacturer's instructions, TRIzol reagent (Servicebio, Wuhan, China) was applied to extract total RNA from the samples. cDNA was synthesized using a cDNA Reverse Transcription Kit (Servicebio®RT First-Strand cDNA Synthesis Kit, China). Then, qRT-PCR was performed using 2× SYBR Green qPCR Master Mix (Low ROX) kit (Servicebio, Wuhan, China). The qPCR condition was set as follows: 95°C, 30 s; 94°C, 5 s; 58°C, 15 s; and 72°C, 10 s; 40 cycles, followed by a 5-minute final extension at 40°C. Relative expression levels of mRNA were calculated using the 2^-*ΔΔ*Ct^ method (Ct of target genes minus the Ct of GAPDH) [28]. The primer sequence of IL-4 is as follows: AGTTCCACAGGCACAAGCAGC (forward) and TCATGATCGTCTTTAGCCTTTCC (reverse).

### 2.7. Western Blotting Analysis

Synovium samples were divided into the M-RA group (*n* = 3), F-RA group (*n* = 3), male No-RA group (*n* = 3), and female No-RA group (*n* = 3). Western blotting analysis was performed in the 4 groups, and the gray values were compared. The proteins were extracted from the samples, and the total protein concentration was determined using the BCA protein concentration determination kit (Multi Sciences, Hangzhou, China). The proteins were separated by 10% SDS-PAGE and transferred to nitrocellulose membranes. The membranes were sealed for 1 hour in 5% skimmed dried milk in a TBST buffer at 37°C and incubated overnight with primary antibody (Sangon Biotech, Shanghai, China) at 4°C. The membranes were then washed in TBST for 3 times and incubated with secondary antibodies at room temperature for 30 minutes. Potent ECL kit (Multi Sciences, Hangzhou, China) was used for chemiluminescence development. The ImageJ (National Institutes of Health, the United States of America) software was used to analyze the optical density of the stripe.

### 2.8. Statistical Analysis

The data were expressed as the mean ± sd, and differences between 2 groups were analyzed with Student's *t*-test. Kruskal-Wallis or Mann–Whitney *U* tests for continuous variables and Chi-square or Fisher's exact tests for categorical variables. *P* < 0.05 was considered statistically significant. Statistical analysis was performed using the Statistical Program for Social Sciences (SPSS) software 19.0 (SPSS Inc., Chicago, IL, USA).

## 3. Results

### 3.1. Differential Expression Genes

**Figure 1:**
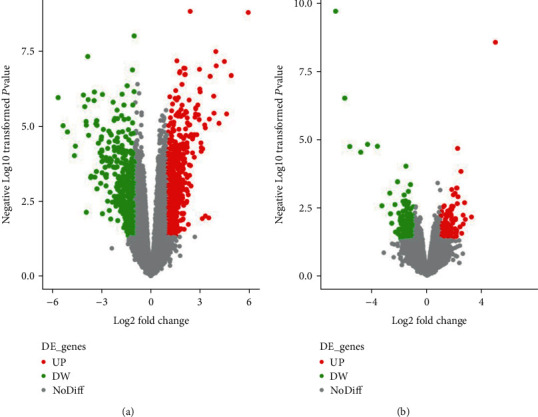
DEG active volcano, screening criteria: *P* < 0.05 and ∣logFC | >1. Red dots represent upregulated genes, and the green dots represent downregulated genes. (a) RA and No-RA groups. (b) M-RA and F-RA groups.

According to GEO2R screening analysis, there were 1230 DEGs (763 upregulated genes and 467 downregulated genes) between the RA group and No-RA group, and 306 DEGs (137 upregulated genes and 169 downregulated genes) between the M-RA and F-RA groups. The volcano maps show that there are significant genetic differences between the members of the two populations. (Figures 1(a) and 1(b)).

### 3.2. GO Function Analysis

**Figure 2:**
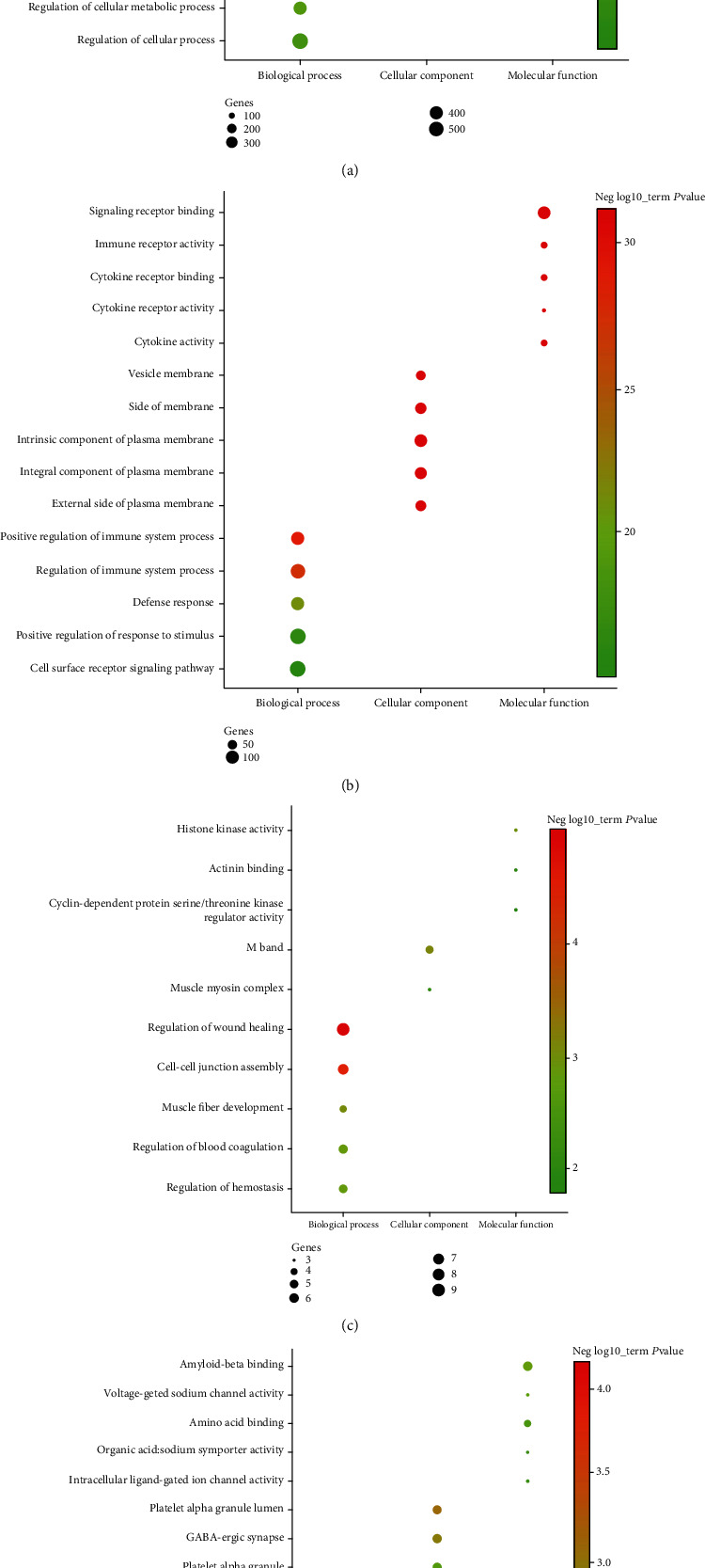
Top 5 GO enrichment analysis of DEGs. GO characteristics of the DEGs are described as biological process, molecular function, and cellular component. The setting conditions were as follows: *P*.adj.value < 0.05, gene count ≥ 3. (a) Upregulated genes between the RA and No-RA groups. (b) Downregulated genes between the RA and No-RA groups. (c) Upregulated genes between the M-RA and F-RA groups. (d) Downregulated genes between the M-RA and F-RA groups.

The upregulated or downregulated genes in the two groups were analyzed by GO enrichment analysis, and GO items were selected according to their *P* < 0.05. The results showed that the upregulated and downregulated genes in the two groups were mainly concentrated in the biological process (Figure 2).

In biological process, the upregulated genes between the RA and No-RA groups participated in regulation of cellular process, positive regulation of biological process, regulation of metabolic process, etc. (Figure 2(a)). Downregulated genes between the RA and No-RA groups were involved in the positive regulation of response to stimulus, cell surface receptor signaling pathway, regulation of immune system process, etc. (Figure 2(b)). Upregulated genes between the M-RA and F-RA groups participated in the positive regulation of small molecule metabolic process, memory, negative regulation of reproductive process, etc. (Figure 2(c)). Downregulated genes between the M-RA and F-RA groups participate in the regulation of wound healing, cell-cell junction assembly, regulation of hemostasis, etc. (Figure 2(d)).

In cellular component, the upregulated genes between the RA and No-RA groups were involved in intracellular organelle, membrane-bounded organelle, plasma membrane, etc. (Figure 2(a)). Downregulated genes between the RA and No-RA groups participated in the intrinsic component of the plasma membrane, integral component of the plasma membrane, side of the membrane, etc. (Figure 2(b)). Upregulated genes between the M-RA and F-RA groups participated in the regulation of M band, muscle myosin complex (Figure 2(c)). Downregulated genes between the M-RA and F-RA groups were involved in platelet alpha granule, platelet alpha granule lumen, GABA-ergic synapse, etc. (Figure 2(d)).

In terms of molecular function, the upregulated genes between the RA and No-RA groups participated in signaling receptor binding, sequence-specific DNA binding, regulatory region nucleic acid binding, etc. (Figure 2(a)). Downregulated genes between RA and No-RA participated in signaling receptor binding, immune receptor activity, cytokine receptor binding, etc. (Figure 2(b)). Upregulated genes between the M-RA and F-RA groups participated in cyclin-dependent protein serine/threonine kinase regulator activity, histone kinase activity, and actinin binding (Figure 2(c)). Downregulated genes between the M-RA and F-RA groups were involved in amyloid-beta binding, amino acid binding, voltage-gated sodium channel activity, etc. (Figure 2(d)).

### 3.3. KEGG Pathway Enrichment Analysis

**Figure 3:**
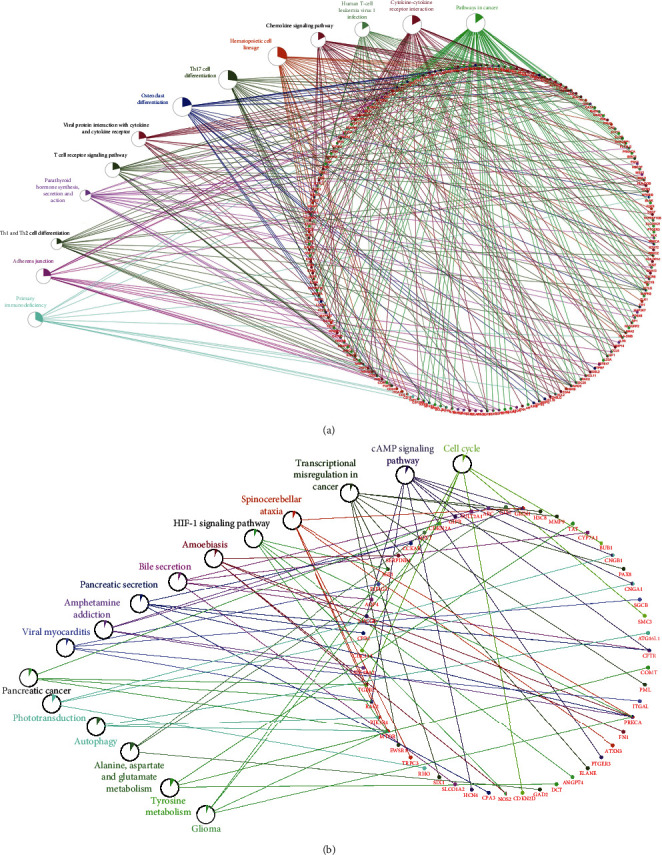
KEGG pathway analysis of DEG. The setting conditions were as follows: *P* < 0.05, gene count ≥ 3. (a) RA and No-RA groups. (b) M-RA and F-RA groups.

The DEGs of the two groups were enriched and showed directly by KEGG pathways. The KEGG pathways involved in DEG between the RA and No-RA groups included pathways in cancer, Cytokine-cytokine receptor interaction, Human T-cell leukemia virus 1 infection, etc. (Figure 3(a)). The KEGG pathways involved in DEG between the M-RA and F-RA groups include cAMP signal transduction pathways, transcriptional disorders in cancer, autophagy, etc. (Figure 3(b)).

### 3.4. PPI Network Construction

**Figure 4:**
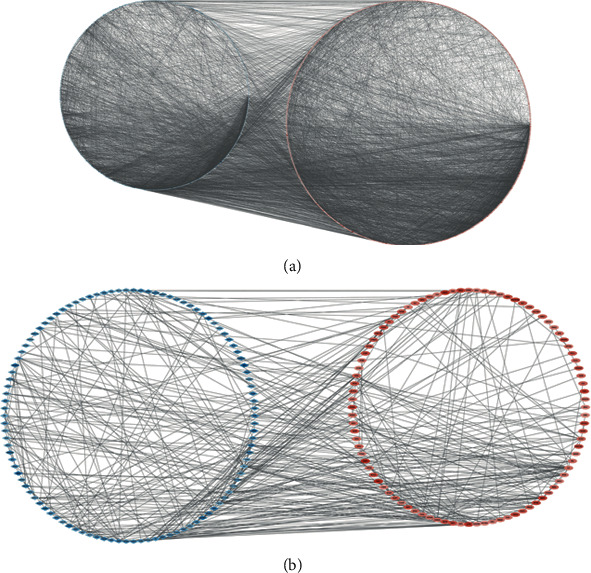
All DEG PPI networks are visualized via Cytoscape. Red balls represent upregulated genes, and blue diamonds represent downregulated genes. (a) RA and No-RA groups. (b) M-RA and F-RA groups.

The interaction between 2 groups of DEGs was evaluated by the STRING database, and PPI network structure was constructed. Data were entered into Cytoscape for visual adjustment. DEG visualization between the RA and No-RA groups has 1140 nodes and 10000 edges (Figure 4(a)). The DEG visualization between the M-RA and F-RA groups has 280 nodes and 462 edges (Figure 4(b)).

### 3.5. Identification of Key Gene

**Figure 5:**
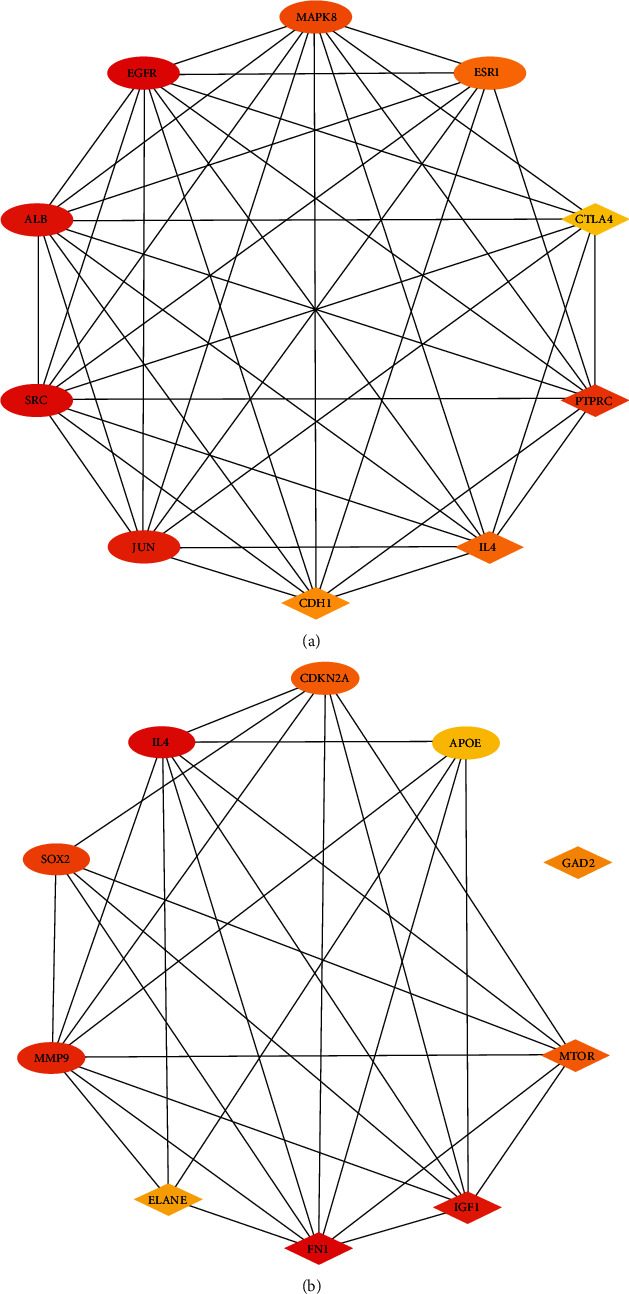
Top 10 genes in degree score from CytoHubba. (a) RA and No-RA groups. (b) M-RA and F-RA groups. The balls represent the upregulated genes, and the diamonds represent the downregulated genes.

**Figure 6:**
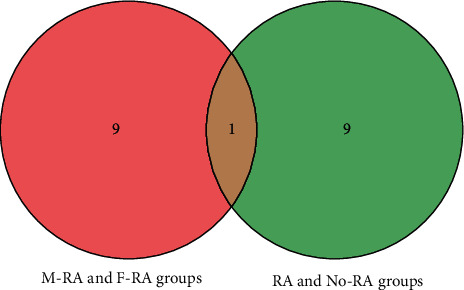
Intersection of 2 sets of top 10 hub genes.

**Figure 7:**
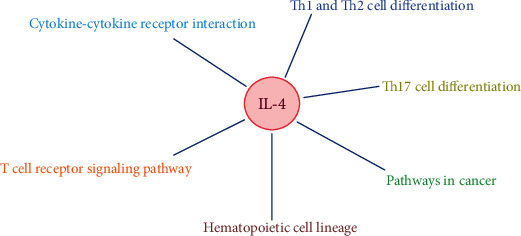
The correlation network between the key gene and KEGG pathways.

CytoHubba revealed 2 sets of the top 10 hub genes (Figures 5(a) and 5(b)). Interleukin-4 (IL-4), as a key gene, was located at the intersection of two sets of HUB genes (Figure 6). The expression of IL-4 in the RA group was lower than that in the normal group. The expression of IL-4 in the F-RA group was lower than that in the M-RA group. The pathways involved in IL-4 between the RA and No-RA groups included cytokine-cytokine receptor interaction, Th1 and Th2 cell differentiation, Th17 cell differentiation, T cell receptor signaling pathway, pathways in cancer, and hematopoietic cell lineage. (Figure 7).

### 3.6. Quantitative Real-Time PCR and Western Blot Analyses

**Figure 8:**
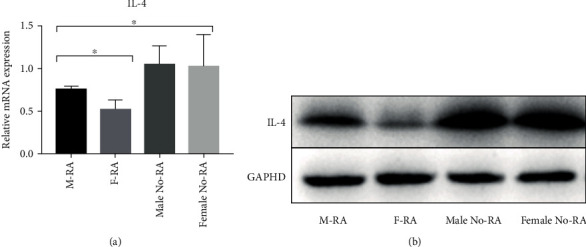
The expression of IL-4 in the RA group was lower than that in the No-RA group (*P* < 0.05). The expression of IL-4 in the F-RA group was lower than that in the M-RA group (*P* < 0.05). (a) Quantitative real-time PCR. (b) Western blot analysis. ^∗^*P* < 0.05.

QPCR analysis showed that the expression of IL-4 in the RA group was lower than that in the No-RA group (*P* < 0.05, *t* = −5.859). The expression of IL-4 in the F-RA group was lower than that in the M-RA group (*P* < 0.05, *t* = −2.793) (Figure 8(a)). Similarly, Western blot analysis showed that the expression of IL-4 in the F-RA group was the lowest, followed by the M-FA group. (Figure 8(b)).

## 4. Discussion

Gender differences in the epidemiological and clinical manifestations of RA have been confirmed [15–22]. Some studies have indicated that the environment, sex hormones, and female menstrual cycle may be related to the gender bias associated with RA [29–31]. Cutolo et al. [32] found that the increase of estrogen level and the decrease of androgen level in the RA synovium seem to play an important role in local immune inflammatory response. Da Silva and Hall [33] argued that although some studies have shown that sex hormones can interfere with many hypothesized processes in the pathogenesis of RA, including immune regulation, inflammatory mediators, interaction of cytokine system, and direct influence of cartilage, trials of sex hormones for potential treatment of rheumatoid arthritis are limited. On the contrary, Silman and Pearson [34] found that female sex hormones may play a protective role in RA. Alpizar-Rodriguez et al. [31] believed that although female sex hormones are related to the development of RA, the complex changes of sex hormones in women's lives cannot fully explain the development process and clinical characteristics of RA. And the effect of sex hormone replacement therapy on RA is not clear. The effect of estrogen on RA is still controversial. In fact, the development of RA may be affected by many factors. Sex hormones and the environment are one of the risk factors for RA [30, 35]. However, RA showed an obvious genetic trend [11, 12]. Therefore, in-depth study from the perspective of the gene is conducive to finding the root cause of the gender difference in RA. We carried out bioinformatics analysis of DEGs according to the gender differences of (RA vs. No-RA group and M-RA vs. F-RA group) and found that the two groups had a common key gene: IL-4. The expression of IL-4 was lower in the RA group, and the expression in the female RA group was lower than that in the male RA group. This suggested that IL-4 may be a potential gene responsible for the pathogenesis of RA and leads to differences in RA between women and men. In addition, it was also found that there was no significant difference in the expression of IL-4 between men and women in the No-RA group (*P* > 0.05). This further confirms that there was a significant gender difference in the expression of IL-4 only in patients with RA. IL-4 leads to the development of RA through the pathways of cytokine-cytokine receptor interaction, Th1 and Th2 cell differentiation, Th17 cell differentiation, T cell receptor signaling pathway, pathways in cancer, and hematopoietic cell lineage and shows the characteristics of female dominance.

RA is a chronic autoimmune disease characterized by the accumulation of inflammatory cells in the synovium and destruction of joints. Some cytokines play a role by promoting inflammation and inducing cartilage degradation. On the contrary, other cytokines mainly play an anti-inflammatory role. The imbalance between the proinflammatory and anti-inflammatory cytokines is the cause of chronic joint damage in RA [36–39]. The protein encoded by IL-4 is a multipotent cytokine produced by activated T cells, which is considered to be an anti-inflammatory cytokine, and can inhibit excessive inflammatory response and counteract the harmful effects of proinflammatory cytokines [37, 40, 41]. In addition, IL-4 also protects synovial cells from apoptosis, inhibits bone resorption, and protects the cartilage tissue from injury [42–46].

Isomäki and Punnonen [36] indicated that in the active stage of RA, the level of anti-inflammatory cytokine IL-4 is too low, so it may not be able to counteract the harmful effects of proinflammatory cytokines. Taki et al. [44] found that IL-4 may increase the probability of synovial dilatation through antiapoptosis. Krabben et al. [47] indicated that the changes of IL-4 and IL-4R genes are closely related to the severity of joint injury in RA. Pawlik et al. [48] found that IL-4 promoter polymorphism may be a genetic risk factor for the severity of RA. Sun YH et al. [49] believed that the gene polymorphism of IL-4 may be closely related to the occurrence of RA. Carrying T allele will greatly increase the risk of RA and reduce the mRNA expression of IL-4. Müller-Ladner et al. [50] indicated that IL-4 STAT is involved in the key pathogenesis of the RA synovium, and the IL-4 STAT-dependent pathway plays a role in the early and late stages of the disease and may contribute to the inhibitory immune mechanism of the RA synovium. Harada et al. [51] pointed out that IL-4 may downregulate rheumatoid inflammation by inducing 15-LOX and its metabolites. Miyata et al. [52] believed that the significant decrease in the synthesis of mRNA of IL-4 and IL10 is related to the progress and activity of RA, so the possibility of using IL-4 and IL10 to treat progressive or intractable RA should be considered. Morita et al. [38] found that both IL-4 and IL10 have the therapeutic potential to regulate the disease activity mediated by proinflammatory cytokines in RA. Steen-Louws et al. [53] developed a newly developed fusion protein of IL-4 and IL10, which can transfer a variety of proinflammatory pathways to immune regulation and inhibit proinflammatory activity in arthritis models. In addition, IL-4 mediates and regulates a variety of human host responses, such as allergy, antiparasite response, tumor immunity, and acute inflammation [54–56].

We believe that IL-4 is an anti-inflammatory cytokine. The balanced secretion of IL-4 and proinflammatory cytokines is an important condition for the normal physiological activity of joints. As the hub of the activity, the joint secretes synovial fluid and various cytokines, which can effectively cushion the stress, reduce the wear of the articular surface, and prevent the destruction of the cartilage tissue. Under normal circumstances, sufficient expression of IL-4 can effectively inhibit mild inflammation and compensate for mechanical injury caused by joint activity. Low expression of IL-4 may lead to the destruction of joint homeostasis and relative overexpression of proinflammatory cytokines, resulting in joint inflammation and bone damage. This may be a key factor leading to rheumatoid arthritis. The expression of IL-4 in women is lower than that in men, so the proportion of women in RA population is higher, and the symptoms of joint inflammation and damage are more severe than men.

## 5. Conclusion

The topic of gender differences in RA deserves further discussion. The low expression of IL-4 may be the susceptibility factor of RA, which promotes the progress of RA through the pathway of cytokine-cytokine receptor interaction, Th1 and Th2 cell differentiation, Th17 cell differentiation, etc, and carries the characteristics of female dominance.

### 5.1. Future Perspective

RA is a chronic inflammatory disease characterized by symmetrical peripheral polyarthritis, which seriously affects the quality of life of patients. A large number of studies have shown that RA has the characteristics of gender dimorphism in clinical manifestations. Its mechanism has not been fully elucidated. This study reveals the key genes and related pathways that may lead to gender differences in the pathogenesis of RA and provides a new understanding of the pathogenesis of RA. Therefore, gender dimorphism should be considered in the research and treatment of RA. This discovery will contribute to a further comprehensive understanding of the biological characteristics of RA and provide new molecular targets for individualized therapy.

### 5.2. Summary Points

- The gene expression data of GSE55457 were analyzed by bioinformatics method to find out the DEGs and related pathways between the RA and No-RA group and male and female RA groups
- We found that they share common hub genes
- We identified the key genes and related pathways that lead to gender differences from the network between hub genes and KEGG pathways
- By combining with quantitative real-time PCR, Western blot analysis, and previous studies, we found that IL-4 may be the key gene leading to gender differences in RA
- Our study provides new insights into the pathogenesis of RA and a new molecular target for individualized therapy

## Data Availability

The datasets supporting the conclusions of this article are available in the GEO repository, https://www.ncbi.nlm.nih.gov/geo/.

## Abbreviations

RA: Rheumatoid arthritis
DEGs: Differentially expressed genes
M-RA: Male RA
F-RA: Female RA
FC: Fold-change
GO: Gene ontology
KEGG: Kyoto Encyclopedia of Genes and Genomes
PPI: Protein-protein interaction
SPSS: Statistical Program for Social Sciences
IL-4: Interleukin-4.
